# A Pilot, Prospective, Randomized-Controlled Study to Evaluate the Efficacy and Safety of Arsha Hita™ in the Treatment of Anal Fissures

**DOI:** 10.7759/cureus.37531

**Published:** 2023-04-13

**Authors:** Gopikrishna B. J., Sahanasheela K. R., Suhas K Shetty, Prasanna N Rao, Sangam Narvekar, Megha Nalawade, Mukesh B Chawda, Kruttika R Chitnis, Rajmohan Seetharaman, Raakhi K Tripathi

**Affiliations:** 1 General Surgery (Shalya Tantra), Sri Dharmasthala Manjunatheshwara College of Ayurveda and Hospital, Hassan, IND; 2 Psychiatry (Manas Rog), Sri Dharmasthala Manjunatheshwara College of Ayurveda and Hospital, Hassan, IND; 3 Medical Services, Shree Dhootapapeshwar Limited, Mumbai, IND; 4 Clinical Research, Shree Dhootapapeshwar Limited, Mumbai, IND; 5 Pharmacology and Therapeutics, Seth Gordhandas Sunderdas Medical College and King Edward (VII) Memorial Hospital, Mumbai, IND

**Keywords:** vedic medicine, physician’s global impression, participant’s global impression, visual analogue scale, lidocaine plus nifedipine, assessor-blind study, parallel group, two-arm, active-controlled, ayurveda

## Abstract

Introduction

Anal fissures are tears in the anal canal that cause pain, bleeding, and spasms. They can be treated with non-operative options such as sitz baths, local anesthetics, topical nitrates, oral fiber, and calcium channel blockers, but some patients require surgery. Topical nitrates have side effects such as severe headaches, while topical calcium channel blockers can cause itching. There is a need to explore alternative treatments with fewer side effects. This proof-of-concept pilot study aimed to compare the efficacy and safety of a combination of Arsha Hita™ tablets and ointment (Shree Dhootapapeshwar Limited, Mumbai Maharastra, India) (test treatment) with a combination of lidocaine 1.5% w/w + nifedipine 0.3% w/w cream for local application and Isabgol powder (6 g) orally as an active comparator (standard treatment), which is the standard treatment of anal fissures as per the Association of Colon and Rectal Surgeons of India (ACRSI) guidelines.

Methodology

This study was a single-center, prospective, randomized-controlled study conducted in Karnataka, India. Participants were screened for anal fissures and randomized to receive either standard treatment (Group A) or test treatment (Group B) for 14 days, and were re-evaluated after two, four, and six weeks. The study assessed signs and symptoms related to anal fissures, such as pain post-defecation on Visual Analog Scale (VAS), bleeding per anus grading, wound healing grade, stool consistency, and stool frequency. Compliance, inter-current illness, and concomitant therapy were noted at each visit. The study used independent sample t-tests to compare variables at baseline and chi-square or Fisher's exact tests to compare the number/proportion of participants achieving primary and secondary endpoints. Mann-Whitney U test was used to compare median composite scores at baseline and Visit 4, and Friedman's two-way analysis of variance was used to compare median composite scores across the four visits (p < 0.05 was considered significant). Descriptive analysis was used to assess VAS, bleeding, and healing grades.

Results

The study included 53 participants with anal fissures, of which 25 out of 27 allocated in Group A (two drop-outs) received standard treatment, and all 26 allocated in Group B received Arsha Hita treatment. At the end of the study, 11 participants in Group B achieved a 90% reduction in composite scores compared to only three patients in Group A (p<0.05). Both groups showed improvement in pain on defecation, severity of bleeding, healing of anal fissure wound, and participant's and physician's global impression score. Group B had significantly better results in terms of VAS score, resolution of per-anal bleeding, and physician's global impression score (p<0.05). There were no adverse events in either group during the six-week treatment period.

Conclusion

The pilot study provides evidence that the combination of Arsha Hita tablets and Arsha Hita ointment may be more effective and safer for treating anal fissures than the standard treatment. The test treatment group experienced greater pain relief, complete resolution of per-anal bleeding, and better global impression scores than the standard treatment group. These findings suggest the need for further research through larger, randomized controlled trials to determine the efficacy and safety of Arsha Hita in treating anal fissures.

## Introduction

An anal fissure is a tear that happens in the anal canal, starting from the dentate line and extending up to the anal verge. Symptoms of anal fissures include pain during or after defecation, anal bleeding, and spasms of the anal sphincter. This condition typically affects middle-aged and young patients, with an average onset age of 39.9 years [1]. Anal fissures occur in approximately one in 350 adults, and the most prevalent symptoms are pain and bleeding [2].

Anal fissures are classified as either acute or chronic, depending on the duration of symptoms. Non-operative treatments for anal fissures include sitz baths, local anaesthetics, topical nitrates, oral fibre intake, laxatives, and oral or topical calcium channel blockers (CCBs). However, some patients may not experience symptom relief with these options and require surgery. Topical nitrates may cause severe headaches in up to 20% of patients, while itching is the primary side effect of topical CCBs in about 15% of patients [3]. Patients with chronic anal fissures who undergo surgery may be at high risk for anal incontinence, and post-surgical recurrence of anal fissures is not uncommon [1]. Therefore, alternative treatment approaches with minimal side effects may be worth considering for individuals with anal fissures.

Arsha Hita™ tablets and ointment (Shree Dhootapapeshwar Limited, Mumbai, India) are Ayurvedic formulations that are commercially available in the Indian market. Arsha Hita tablets contain Sarja or Indian Copal Tree (*Vateria indica*), Arishtak or Soapnut tree (*Sapindus trifoliatus*), and Soorana or Elephant foot yam (*Amorphophallus campanulatus*), all of which have anti-inflammatory properties. Arishtak and Soorana have been specifically noted for their anti-inflammatory actions. Sarja, on the other hand, contributes to the wound-healing action [4-6]. On the other hand, Arsha Hita ointment is formulated with Tila Taila or sesame oil (*Sesamum indicum*), which has wound-healing and analgesic properties [7]. Karpoora or camphor (*Cinnamomum camphora*) is another key ingredient that provides analgesic and anti-pruritic benefits [8]. Additionally, Madhoochchhishta (Bee's wax) contains anti-inflammatory, wound healing, and styptic properties [9]. Similar to Arsha Hita tablets, Arsha Hita ointment also contains Sarja, which further helps speed up wound healing [6].

Arsha Hita tablets and ointment are used to manage the symptoms of bleeding, pain, anal discomfort, and inflammation associated with anal fissures and hemorrhoids. These formulations have been traditionally used by Ayurved practitioners for managing piles/hemorrhoids and anal fissures. However, there was a need to confirm their efficacy and safety in human subjects to validate their use.

Isabgol powder, which is derived from the seeds of the *Plantago ovata* plant, is a soluble fiber that can absorb water and produce a gel-like substance in the gastrointestinal tract. This substance can help soften stools and alleviate constipation. Isabgol powder is frequently recommended for individuals who have trouble passing stools or experience irregular bowel movements. Additionally, due to its laxative properties, it may also be used in the treatment of anal fissures to help ease bowel movements and reduce discomfort during defecation. This was used as an adjuvant to the standard treatment in the study [10].

Topical agents used in combination for managing anal fissures have not been found to provide complete and satisfactory relief while also avoiding adverse effects [3]. Arsha Hita tablets, used systemically, and Arsha Hita ointment, applied topically, could be effective conservative management options for fissures. Hence, this proof-of-concept pilot study aimed to compare the efficacy and safety of a combination of Arsha Hita tablets and ointment with the combination of lidocaine 1.5% w/w + nifedipine 0.3% w/w cream for local application, and Isabgol powder (6 g) orally, which is the standard treatment for anal fissures and serves as an active comparator as per Association of Colon and Rectal Surgeons of India (ACRSI) Guidelines [11].

## Materials and methods

Study design

The trial was conducted in the Outpatient Department (OPD) and Inpatient Department (IPD) of Shalya Tantra, Sri Dharmasthala Manjunatheshwara College of Ayurveda and Hospital, Karnataka, India, as a single centre, prospective, randomized, active-controlled, two-arm, parallel-group, assessor-blind study and included patients with anal fissures. The study was initiated after the approval by the Ethics Committee of Sri Dharmasthala Manjunatheshwara College of Ayurveda and Hospital (approval number: SDM/IEC/1072018-19) and the subsequent receipt of Clinical Trials Registry-India Approval (CTRI/2020/02/023229) on February 10, 2020. The total duration of the study was 12 months. 

Study participants, randomization, and visit schedule

A surgeon not affiliated with the study team evaluated individuals seeking treatment for anal fissure at the Shalya Tantra OPD. The study included patients of either gender, aged 18-60 years, who had a confirmed diagnosis of anal fissure, a composite score of more than 4, and no prior treatment or only conservative treatment (such as sitz bath, Isabgol powder, or high-fiber diet) in the past month. Patients with anal fistulas due to secondary causes (such as Crohn's disease, anal suppuration, or abscesses), anal or perianal malignancies, more than three recurrences of anal fissure after medical or surgical intervention, per-anal bleeding due to secondary causes (such as hemorrhoids, abscesses, cancers, inflammatory bowel disease, or active infections), or major systemic illnesses were not included in the study. Patients who had taken oral or topical vasodilators (such as calcium channel blockers, minoxidil, hydralazine, nitroglycerine, lignocaine, and steroids) in the past month or herbal medications in the past three months were also excluded. To ensure an equal number of participants in each group, an open list of random numbers was utilized to assign eligible participants to one of two treatment groups. Each participant's involvement in the study lasted for eight weeks, as per the detailed visit schedule provided in Table 1. At each follow-up visit, study personnel recorded all variables specified in the protocol. An independent surgeon, not affiliated with the study team, assessed the study variables. If a participant withdrew or dropped out of the study, the investigator clinically evaluated them and recorded the reason for discontinuation in the Case Record Form.

**Table 1:** Visit schedule of the study participants Sr. No.: Serial Number; HIV: Human Immunodeficiency Virus; HBsAg: Hepatitis B Surface Antigen; HCV: Hepatitis C Virus; Hb/CBC: Hemoglobin/Complete Blood Count; RBS: Random Blood Sugar; SGOT: Serum Glutamic Oxaloacetic Transaminase; SGPT: Serum Glutamic Pyruvic Transaminase; Urine R and M: Urine Routine and Microscopy; and VAS: Visual Analog Scale Variables assessed during the Baseline Visit were for screening and those assessed on Visit 1, Visit 2, Visit 3, and Visit 4 were for efficacy and safety evaluation.

Sr. No.	Assessment	Baseline (Screening) Visit	Visit 1 (Week 0)	Visit 2 (Week 2)	Visit 3 (Week 4)	Visit 4 (Week 6)
		Day -3 to Day -7	Day 0	Day 14 ± 2	Day 28 ± 2	Day 42 ± 2
1	Informed consent	Yes	No	No	No	No
2	History	Yes	No	No	No	No
3	General Examination	Yes	No	Yes	Yes	Yes
4	Systemic Examination	Yes	No	Yes	Yes	Yes
5	Blood Investigations					
5a	HIV	Yes	No	No	No	No
5b	HBsAg	Yes	No	No	No	No
5c	HCV	Yes	No	No	No	No
5d	Hb/CBC	Yes	No	No	No	Yes
5e	RBS	Yes	No	No	No	Yes
5f	Serum Creatinine	Yes	No	No	No	Yes
5g	Serum Bilirubin, SGOT, and SGPT	Yes	No	No	No	Yes
5h	Urine R and M	Yes	No	No	No	Yes
5i	Urine Pregnancy Test	Yes	No	No	No	No
6	Randomization	No	Yes	No	No	No
7	Medication Dispensing	No	Yes	No	No	No
8	Medication Compliance	No	No	Yes	Yes	Yes
9	VAS for pain assessment	Yes	No	Yes	Yes	Yes
10	Bleeding per anus grading	Yes	No	Yes	Yes	Yes
11	Anal fissure wound evaluation	Yes	No	Yes	Yes	Yes
12	Participants and Physician’s global impression of improvement	No	No	No	No	Yes
13	Adverse Events (Local and Systemic)	No	Yes	Yes	Yes	Yes

Study interventions

Participants who met the eligibility criteria were randomly divided into two groups, Group A (Standard Treatment Group) and Group B (Test Treatment Group), in a 1:1 allocation and received treatment for six weeks. Participants in Group A were given lidocaine 1.5% w/w + nifedipine 0.3% w/w cream 1 g (equivalent to two fingertip units (FTU)) to be applied locally three times daily for six weeks, along with one sachet of Isabgol powder (6 g) to be consumed orally with 200 ml lukewarm water at bedtime daily. Participants in Group B were given Arsha Hita ointment 1 g (equivalent to 2 FTU) to be applied locally four times daily (especially before and after evacuation) for six weeks, along with two tablets of Arsha Hita tablets to be taken orally twice a day with lukewarm water. Conservative treatment, including sitz bath and high-fiber diet, was common to both groups. All participants provided written informed consent before the trial began.

Shree Dhootapapeshwar Limited, Mumbai, Maharashtra provided the test treatment, Arsha Hita tablets, and Arsha Hita ointment, and the standard treatment, lidocaine 1.5% w/w + nifedipine 0.3% w/w cream and oral Isabgol powder (6g), which were provided in a kit in a plastic box. The test treatment was manufactured according to Good Manufacturing Practices (GMP) standards. The lidocaine 1.5% w/w + nifedipine 0.3% w/w cream was procured from the local market, and the oral Isabgol powder (6g) was procured from Om Pharmaceuticals Limited (Bengaluru, Karnataka, India). All kits and medications had the study code, expiry date, name of the sponsor, batch number of the drug, storage conditions, and the statement “For Clinical Trial purpose only”.

The composition of each Arsha Hita coated tablet was Sarja 200 mg (*Vateria indica*), Arishtaka 100 mg (*Sapindus trifoliatus*), and Soorana 50 mg (*Amorphophallus campanulatus*). The composition of each 10 g Arsha Hita ointment was Tila Tailum 6.420g (Oil of *Sesamum indicum*), Sarja 1.600 g (*Vateria indica*), Bhimseni Karpoora 0.300 g (*Cinnamomum camphora*), and *Madhoochchhishta* q.s. (Bee’s wax).

Efficacy and safety endpoints

Table 2 shows the efficacy variables, which include the primary endpoint of composite score in Group A compared to Group B. The secondary endpoints include the number of participants with a global impression score greater than 6, complete resolution of fissures (physician's global impression score = 0), reduction of VAS score to less than 2, complete healing of anal fissure wounds (grade III wound healing), resolution of per-anal bleeding during defecation (grade 0), and improvement in serum hemoglobin levels. The composite score, ranging from 0 to 12, was obtained by combining the VAS score (ranging from 0 to 10) for pain during defecation and the grading score for bleeding per anus (ranging from 0 to 2). The participant’s global impression of improvement was assessed using a second-point Likert scale ranging from 1 to 7, where 1 indicated a significant decline and 7 indicated a significant improvement. Similarly, the physician's global impression score was categorized as completely resolved (score=0), improved (score=1), or failure (score=2). The healing of fissure wounds was graded into three categories, with Grade III indicating complete epithelial coverage of the wound. To assess safety, general and systemic examinations, adverse event recording, and monitoring of blood and urine investigations were conducted. Safety endpoints included the number of participants with adverse events and abnormal physical examination findings.

**Table 2:** Efficacy variables and scores Sr. No.: Serial Number, VAS: Visual Analogue Scale

Sr. no.	Efficacy Variable	Scores/Grades	Interpretation
1	Visual Analogue Scale (VAS)	Score 0	Minimum: No pain to patient on defecation
		Score 10	Maximum: Unbearable pain to patient while passing stools.
2	Bleeding per anus	Score 0	Grade 0: No sign of hemorrhage upon defection
		Score 1	Grade 1: Sometimes a sign of hemorrhageupon defection
		Score 2	Grade 2: A sign of hemorrhage upon defection
3	Composite Score	Score 0	Minimum score
		Score 10	Maximum score
4	Wound healing	Grade I	Severe and fresh wound with inflammation
		Grade II	Granulation tissue on wound
		Grade III	Completed layer of epithelia covering the wound
5	Stool Consistency	Score 0	Soft
		Score 1	Firm
		Score 2	Hard
6	Stool Frequency	Grade 0	Regular
		Grade 1	Irregular
7	Hemoglobin levels	-	Based on Complete Blood Count
8	Participant’s global impression of improvement	Score 1	Substantially worse
		Score 2	Moderately worse
		Score 3	Slightly worse
		Score 4	No change
		Score 5	Slightly improved
		Score 6	Moderately improved
		Score 7	Substantially improved
9	Physician’s global impression of improvement	Score 0	Completely Resolved: No signs and symptoms at the end of therapy, i.e., VAS=0, Bleeding grade=0, wound healing grade III, stool consistency score 0 etc.
		Score 1	Improved: Reduction of signs and symptoms but not complete relief.
		Score 2	Failure: Signs and symptoms as persisted, severe as, or worse than, those before treatment.

Sample size and statistical analysis

Since this was a proof-of-concept pilot study, a formal sample size calculation was not performed. The study aimed to evaluate 60 participants with anal fissures at the end of the study period, with equal numbers (30 participants) in each group. Independent sample t-tests were used to compare baseline variables such as age, hemoglobin levels, RBC, WBC, neutrophils, lymphocytes, and random blood sugar (RBS). Chi-square test/Fisher's exact test was employed to compare the number/proportion of participants who achieved the primary and secondary endpoints. Median composite scores at the screening (baseline) visit and Visit 4 were compared using the Mann-Whitney U test. Friedman’s two-way analysis of variance was utilized to compare median composite scores across the four visits. Descriptive analysis was employed to determine the percentage of participants with various points on VAS and various grades used for assessing bleeding and healing. Summary statistics were used for all other assessments. The study used a significance level of P < 0.05. The data were analyzed using IBM SPSS Statistics for Windows, Version 25.0 (Released 2017; IBM Corp., Armonk, New York, United States).

## Results

**Figure 1:**
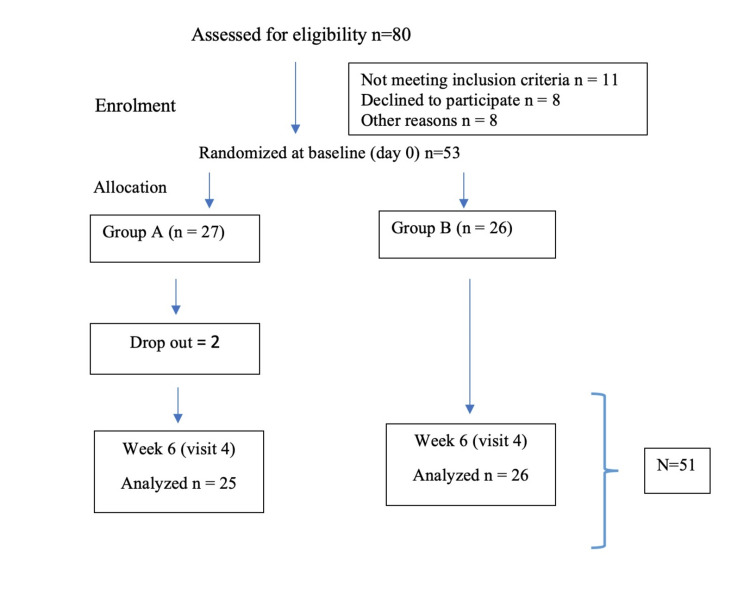
CONSORT Flow diagram CONSORT: Consolidated Standards Of Reporting Trials

A total of 53 participants were recruited, with 27 assigned to Group A (Standard Treatment Group), which received lidocaine 1.5% w/w + nifedipine 0.3% w/w cream and oral Isabgol powder (6 g), and 26 assigned to Group B (Test Treatment Group), which received Arsha Hita tablets and Arsha Hita ointment. However, only 51 participants completed the study, with 25 in Group A and 26 in Group B, as shown in the Consolidated Standards Of Reporting Trials (CONSORT) flow diagram in Figure 1. The baseline parameters of the study participants can be found in Table 3.

**Table 3:** Baseline parameters at screening visit and visit 4 SGOT: Serum Glutamic Oxaloacetic Transaminase; SGPT: Serum Glutamic Pyruvic Transaminase Tests used: Unpaired t Test (Group A Vs Group B at baseline and Visit 4), and Paired t Test (Baseline visit Vs Visit 4 for Group A and Group B) The only significant difference found between Group A and Group B was in their RBC count (p=0.001 at baseline and p=0.001 at visit 4) and RBS level (p=0.003 at baseline), but both parameters were still within the normal range and therefore not clinically significant. For reference, the normal range for RBC count is typically 4.5-5.5 million/mm^3^, and the normal range for RBS is typically 70-99 mg/dL but can increase to 100-125 mg/dL in prediabetes.

Variables	Baseline visit (Mean ± SD)		Visit 4 (Mean ± SD)	
	Group A (n =25)	Group B (n =26)	Group A (n =25)	Group B (n =26)
Age (years)	34.4 ± 10.04	30.42 ± 6.63		
Haemoglobin (g/dl)	12.13 ± 1.44	12.4 ± 1.41	12.52 ± 1.28	13.06 ± 1.11
RBC (million/mm^3^)	4.12 ± 0.62	4.95 ± 0.71	4.29 ± 0.6	5.37 ± 0.66
WBC (cells/mm^3^)	8376 ± 1730	8077 ± 1949	7828 ± 1380	7863 ± 1511
Neutrophils (%)	56.76 ± 7.73	56.76 ± 7.26	57.64 ± 7.7	58.35 ± 7.1
Lymphocytes (%)	36.76 ± 6.83	34.77 ± 6.72	35.64 ± 6.8	36.77 ± 6.68
Random blood sugar (mg/dl)	114.46 ± 22.75	98.44 ± 12.1	108.24 ± 19.15	103.15 ± 8.47
Creatinine (mg/dl)	0.81 ± 0.09	0.8 ± 0.12	0.79 ± 0.11	0.83 ± 0.13
Serum bilirubin (mg/dl)	0.82 ± 0.13	0.8 ± 0.2	0.72 ± 0.08	0.7 ± 0.1
SGOT (U/L)	33.62 ± 0.98	33.56 ± 1.01	31.00 ± 0.8	30.96 ± 0.8
SGPT (U/L)	33.07 ± 1.38	32.65 ± 1.44	30.96 ± 0.84	30.8 ± 0.9

Comparison of composite scores between and within the two groups

**Figure 2:**
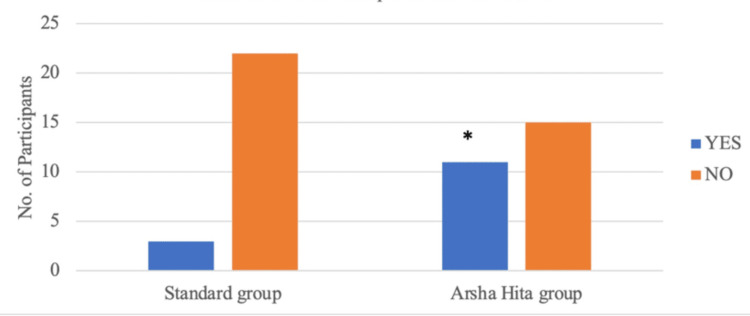
Comparison of participants showing 90% reduction in composite score in both groups in Visit 4 *2-tailed p-value (Fisher’s exact test) is 0.027

At Visit 4 (after six weeks of treatment), three out of 25 participants in Group A and 11 out of 26 participants in Group B demonstrated a 90% reduction in the Composite Score compared to the Baseline (Screening) Visit. The number of participants showing a 90% reduction in the composite score in Group B was significantly higher (p<0.05) than that in Group A, as indicated in Figure 2 and Table 4.

**Table 4:** Comparison of number of patients showing 90% reduction in composite score of both groups at Visit 4

Groups	Number of patients showing 90% reduction at Visit 4		Total
	YES	NO	
Group A (Standard Treatment Group)	3	22	25
Group B (ArshaHita Treatment Group)	11	15	26
Total	14	37	51
2-tailed p-value (Fisher’s exact test)			0.027

**Figure 3:**
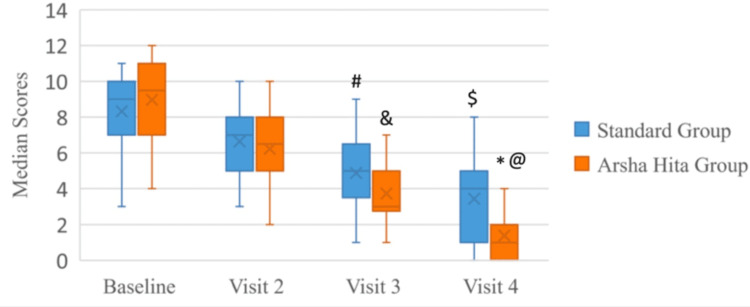
Clustered box-plot of visit-wise median composite scores *p<0.05 at Visit 4 Group B vs Group A: Mann Whitney U test ^@^p<0.001 at Visit 4 vs Baseline Visit (Group B): Friedman test ^$^p<0.001 at Visit 4 vs Baseline Visit (Group A): Friedman test ^&^p<0.001 at Visit 3 vs Baseline Visit (Group B): Friedman test ^#^p<0.001 at Visit 3 vs Baseline Visit (Group A): Friedman test

**Figure 4:**
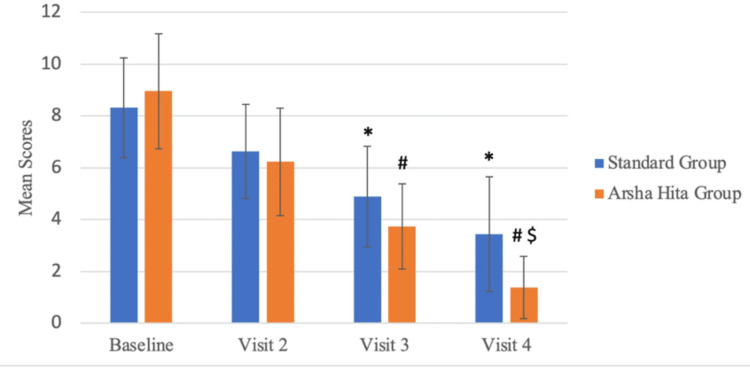
Comparison of mean composite scores between both groups across all visits *p=0.001 at Visit 3 and Visit 4 vs Baseline Visit (Group A): Friedman test ^#^p=0.001 at Visit 3 and Visit 4 vs Baseline Visit (Group B): Friedman test ^$^p=0.001 at Visit 4 Group B vs Group A: Mann-Whitney U test

**Figure 5:**
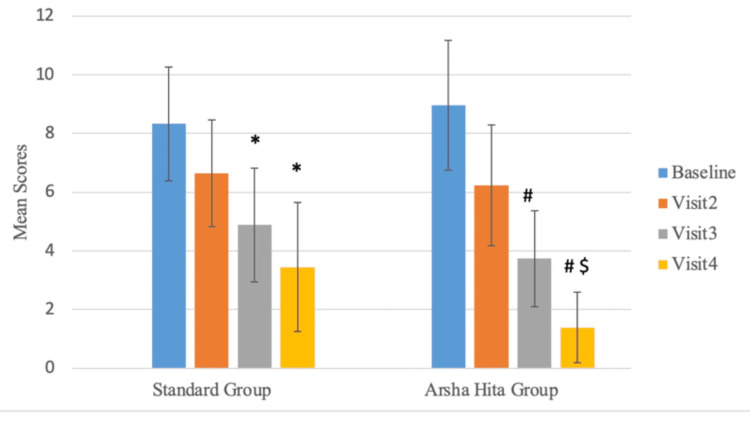
Comparison of mean composite scores within each group across visits *p=0.001 at Visit 4 and Visit 3 vs Baseline Visit (Group A): Friedman test ^#^p=0.001 at Visit 4 and Visit 3 vs Baseline Visit (Group B): Friedman test ^$^p=0.001 at Visit 4 Group B vs Group A: Mann-Whitney U test

The median composite score at the Baseline (Screening) Visit for Group A was 9 with an interquartile range (IQR) of 3, whereas it was 9.5 with an IQR of 4 for Group B (Figure 3, Table 5). There was a decrease in the composite scores at Visit 4 in both groups. The median score for Group A was 4 with an IQR of 4, while the median score for Group B was 1 with an IQR of 2. There was no statistically significant difference in the composite scores at the Baseline (Screening) Visit between Group A and Group B. However, the difference in the decrease in composite scores between the two groups was statistically significant (p<0.001) at Visit 4, as illustrated in Table 6. Figure 4 shows the comparison of mean composite scores between both groups across all visits while Figure 5 shows the comparison within each group.

**Table 5:** Median composite scores at baseline visit and visit 4 IQR: interquartile range

Groups	Composite score at Baseline Median & IQR	Composite score at Visit 4 Median & IQR
Group A (Standard Treatment Group)	9 (7-10)	4 (1-5)
Group B (ArshaHita Treatment Group)	9.5 (7-11)	1 (0-2)
2-tailed p-value (Mann-Whitney U test)	0.229	0.001

**Table 6:** Comparison of mean composite scores between both the groups from baseline (screening) visit to visit 4 *p=0.001 at Visit 3 and Visit 4 vs Baseline Visit (Group A): Friedman test ^#^p=0.001 at Visit 3 & Visit 4 vs Baseline Visit (Group B): Friedman test ^$^p=0.001 at Visit 4 Group B vs Group A: Mann-Whitney U test

Visits	Group A Composite Scores (Mean ± SD)	Friedman test: p value	Group B Composite Scores (Mean ± SD)	Friedman test:p value	Mann Whitney-U test: p value
Baseline	8.32 ± 1.93		8.96 ± 2.22		0.229
Visit 2	6.64 ± 1.82	0.148	6.23 ± 2.07	0.06	
Visit 3	4.88 ± 1.94	0.001*	3.73 ± 1.64	0.001^#^	
Visit 4	3.44 ± 2.2	0.001*	1.38 ± 1.2	0.001^#^	0.001^$^

Secondary endpoints

**Figure 6:**
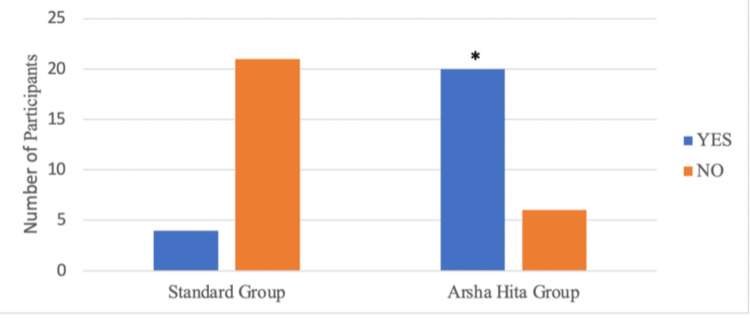
Participants showing a global impression score of >6 at visit 4 *2-tailed p-value (Fisher’s exact test) is 0.001

**Figure 7:**
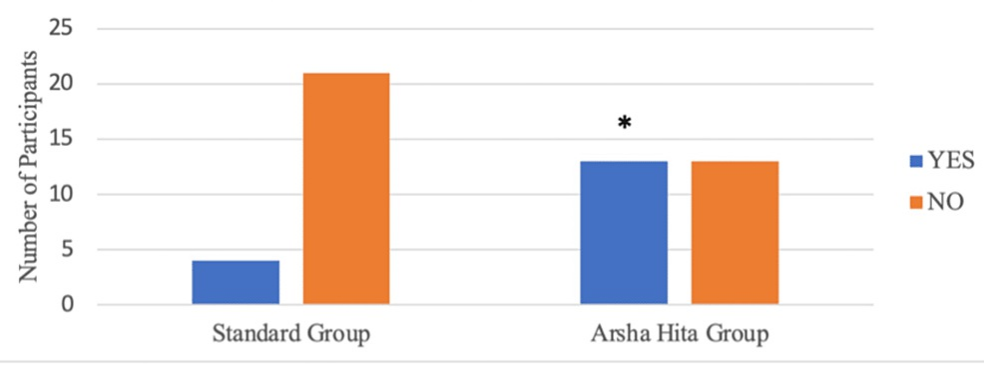
Physicians' global impression score = 0 at visit 4 *2-tailed p-value (Fisher’s exact test) is 0.017

**Figure 8:**
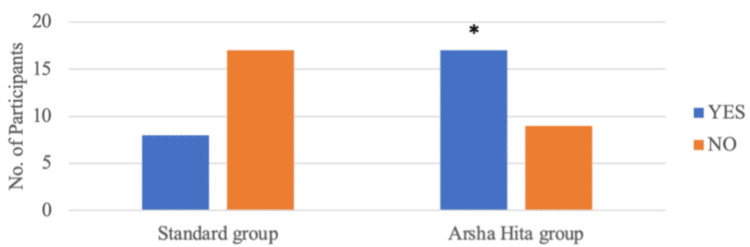
Reduction in the VAS score to < 2 in the two groups at visit 4 VAS: visual analog scale *2-tailed p-value (Chi-square test) is 0.025

During Visit 4, it was observed that four out of 25 participants in Group A and 20 out of 26 participants in Group B had a global impression score greater than 6. Group B had a significantly higher number of participants with a score greater than 6 than Group A (p=0.001) as illustrated in Figure 6 and Table 7. At Visit 4, complete resolution (Physician's global impression score = 0) was noted in four out of 25 participants in Group A and 13 out of 26 participants in Group B. The number of participants with complete resolution was significantly higher in Group B than in Group A (p=0.017) as illustrated in Figure 7 and Table 8. At Visit 4, eight out of 25 participants in Group A and 17 out of 26 participants in Group B had a VAS score of less than 2. The number of participants with a reduction in VAS score less than 2 was significantly higher in Group B than in Group A (p=0.025) as illustrated in Figure 8 and Table 9.

**Table 7:** Participants' global impression score > 6 at visit 4

Groups	Score > 6		Total
	YES	NO	
Group A (Standard Treatment Group)	4	21	25
Group B (ArshaHita Treatment Group)	20	6	26
Total	24	27	51
2-tailed p-value (Fisher’s exact test)			0.001

**Table 8:** Physician’s global impression score = 0 at visit 4

Groups	Score = 0		Total
	YES	NO	
Group A (Standard Treatment Group)	4	21	25
Group B (Arsha Hita Treatment Group)	13	13	26
Total	17	34	51
2-tailed p-value (Fisher’s exact test)			0.017

**Table 9:** Comparison of VAS score < 2 between both groups at visit 4

Groups	VAS < 2		Total
	YES	NO	
Group A (Standard Treatment Group)	8	17	25
Group B (Arsha Hita Treatment Group)	17	9	26
Total	25	26	51
2-tailed p-value (Chi-square test)			0.025

**Figure 9:**
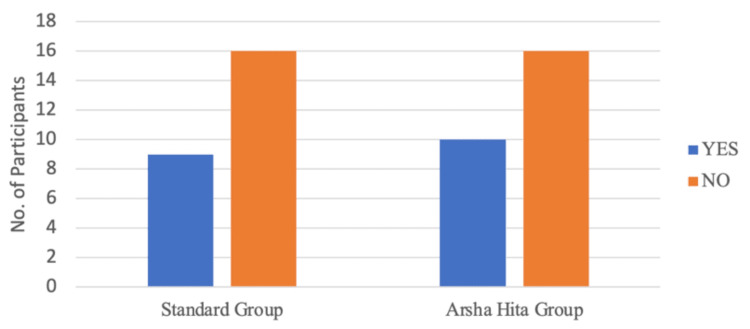
Number of participants showing a complete healing of the anal fissure wounds (Grade III wound healing) at visit 4

**Figure 10:**
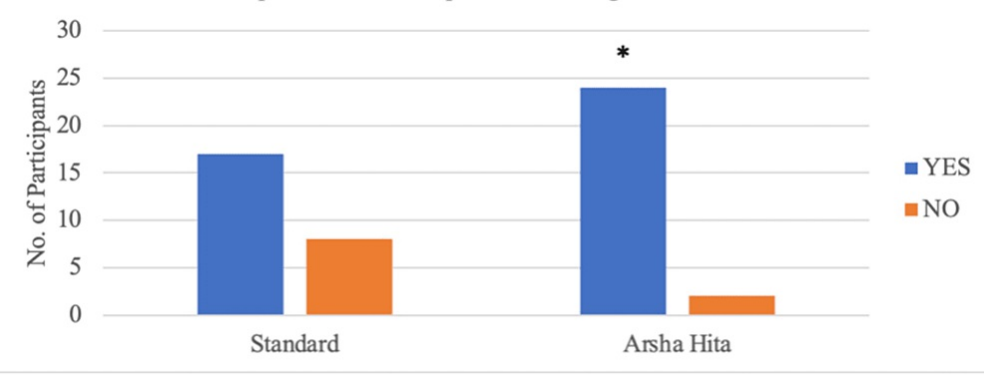
Number of participants showing a complete resolution of per-anal bleeding during defecation (Grade 0) at visit 4 *2-tailed p-value (Chi-square test) is 0.038

At Visit 4, complete wound healing (Grade III) was observed in nine out of 25 participants in Group A and 10 out of 26 participants in Group B, and this outcome was similar (p=0.856) between the two groups as illustrated in Figure 9 and Table 10. A. At Visit 4, complete resolution of per-anal bleeding during defecation (Grade 0) was observed in 17 out of 25 participants in Group A and 24 out of 26 participants in Group B. The number of participants with complete resolution of per-anal bleeding was significantly higher in Group B compared to Group A (p=0.038) as illustrated in Figure 10 and Table 11.

**Table 10:** Grade III wound healing at visit 4

Groups	Grade III wound healing		Total
	YES	NO	
Group A (Standard Treatment Group)	9	16	25
Group B (ArshaHita Treatment Group)	10	16	26
Total	19	32	51
2-tailed p-value (Chi-square test)			0.856

**Table 11:** Complete resolution of per-anal bleeding at visit 4

Groups	Resolution of per-anal bleeding		Total
	YES	NO	
Group A (Standard Treatment Group)	17	8	25
Group B (ArshaHita Treatment Group)	24	2	26
Total	41	10	51
2-tailed p-value (Chi-square test)			0.038

At the Baseline Visit, both Group A and Group B had a mean and median stool consistency score of 2. However, at Visit 4 (after six weeks of treatment), the mean stool consistency score was significantly lower in Group A (0.24 ± 0.44) compared to Group B (0.54 ± 0.508) (p<0.05). The median stool consistency score was 0 for Group A and 1 for Group B at Visit 4. Table 12 shows that there was no significant difference in stool consistency between the two groups at Baseline and Visit 4. At the Baseline Visit, the mean stool frequency scores were 0.72 ± 0.43 and 0.77 ± 0.458 for Group A and Group B, respectively, and the median was 1 for both groups. At Visit 4, the mean stool frequency scores were significantly lower in Group A (0.04 ± 0.2) compared to Group B (0.08 ± 0.27), and the median was 1 for both groups. Table 13 shows that there was no significant difference in stool frequency between the two groups at Baseline and Visit 4.

**Table 12:** Comparison of stool consistency between Group A and Group B at visit 4

Groups	Stool consistency at Baseline		Stool consistency at Visit 4	
	Mean ± SD	Median & (Range, IQR)	Mean ± SD	Median & (Range, IQR)
Group A (Standard Treatment Group)	2 ± 0	2 (0,0)	0.24 ± 0.44	0 (1,1)
Group B (Arsha Hita Treatment Group)	2 ± 0	2 (0,0)	0.54 ± 0.508	1 (1,1)
2-tailed p-value (Mann-Whitney U test)	1.000		0.031	

**Table 13:** Comparison of stool frequency between Group A and Group B at visit 4

Groups	Stool frequency at Baseline		Stool frequency at Visit 4	
	Mean ± SD	Median & (Range, IQR)	Mean ± SD	Median & (Range, IQR)
Group A (Standard Treatment Group)	0.72 ± 0.43	1 (1,1)	0.04 ± 0.2	0 (1, 0)
Group B (Arsha Hita Treatment Group)	0.77 ± 0.458	1 (1,0)	0.08 ± 0.27	0 (1, 0)
2-tailed p value Mann-Whitney U	0.69		0.579	

There was no significant difference in serum hemoglobin levels between the two groups at Baseline Visit (p=0.846) and Visit 4 (p=0.346) as illustrated in Table 14.

**Table 14:** Comparison of hemoglobin levels between the groups at baseline and at visit 4

Groups	Hemoglobin levels at Baseline (Mean ± SD)	Hemoglobin levels at Visit 4 (Mean ± SD)
Group A (Standard Treatment Group)	12.1 ± 1.44	12.5 ± 1.28
Group B (Arsha Hita Treatment Group)	12.4 ± 1.41	13.1 ± 1.11
2-tailed p-value (Independent t-test)	0.846	0.346

Safety endpoints

At Visit 4 (after six weeks of treatment), no adverse events or abnormal findings were observed during the physical examination in any of the participants from both groups.

## Discussion

Anal fissure is one of the most common anorectal problems characterized by anal pain and bleeding per rectum. Acute fissures can often be managed conservatively and typically heal within four to six weeks. However, chronic fissures can persist beyond this time frame. Despite various options for conservative management, some patients continue to experience pain, bleeding, and incomplete wound healing. While there are medications that can alleviate symptoms associated with anal fissures, none of them can completely heal the fissures, and patients may ultimately require surgery [12].

In this study, both the Standard Treatment Group and the Arsha Hita Treatment Group showed a 90% reduction in composite scores after six weeks of treatment (Visit 4). However, a significant difference was observed between the two groups regarding the number of participants who showed a 90% reduction in composite scores. Specifically, 11 out of 26 participants in the Arsha Hita Treatment Group achieved a 90% reduction in composite scores, while only three out of 25 participants in the Standard Treatment Group achieved the same. The composite scores at the baseline visit were not significantly different between the two groups (median 9 vs 9.5), but the reduction in scores after six weeks of treatment (Visit 4) was statistically significant (median 4 vs 1). In addition, there was a significant difference between the two groups in the individual components of the composite scores. Following six weeks of treatment, a significantly greater number of participants in the Arsha Hita Treatment Group experienced pain relief (VAS score <2) and complete resolution of per-anal bleeding (score=0) compared to the Standard Treatment Group.

The number of participants with scores greater than 6 in the participant’s global impression of improvement was significantly greater in the Arsha Hita Treatment Group compared to the Standard Treatment Group after the six-week treatment period (Visit 4). The number of participants with a physician's global impression score of 0 was significantly greater in the Arsha Hita Treatment Group compared to the Standard Treatment Group after six weeks of treatment (Visit 4).

On the evaluation of the healing of fissure wounds after six weeks of treatment (Visit 4), Grade III wound healing was observed in nine out of 26 participants in the Standard Treatment Group, and 10 out of 26 participants in the Arsha Hita Treatment Group. There was no significant difference in the number of participants showing complete wound healing (Grade III) between the two groups. However, there was a significant difference in stool consistency scores at Visit 4 between the two groups. The Standard Treatment Group showed a greater reduction in consistency score, indicating better softening of stools, which may be due to the stool softening action of Isabgol powder [13]. Stool frequency showed no significant difference between the groups at baseline and after six weeks of treatment (Visit 4). Additionally, there was no significant difference in hemoglobin levels between baseline and after six weeks of treatment for both groups.

All baseline and six-week post-treatment investigations, including random blood sugar, total and differential blood counts, serum bilirubin, serum glutamic-pyruvic transaminase (SGPT), and serum creatinine, were within the normal range. Furthermore, none of the participants reported any adverse effects, and no serious adverse events were recorded in either group during the entire study period.

As the primary treatment for anal fissures, non-operative methods that are safe and have fewer side effects are recommended. An assortment of therapies that can provide anti-inflammatory, analgesic, muscle relaxant, laxative, and wound healing effects can be used in an integrated approach to minimize pain and bleeding, as well as to soften stools [1].

In this study, a combination of Arsha Hita tablets and Arsha Hita ointment was found to be an effective initial treatment option for anal fissures that doesn't require surgery. The tablets contain ingredients such as Arishtak (*Sapindus trifoliatus*) and Soorana (*Amorphophallus campanulatus*), which have anti-inflammatory properties, while Sarja (*Vateria indica*) helps with wound healing [4-6]. The Arsha Hita ointment used in the study contains Tila Taila or *Sesame* oil, which is known for its wound healing and analgesic properties. Additionally, it also contains Karpoora, which has analgesic and antipruritic actions [8,14,15]. The third ingredient in the ointment is Bee’s wax, which has anti-inflammatory, wound healing, and styptic effects [9].

The current study suggests that the observed pain relief, resolution of per-anal bleeding, and wound healing in the participants of the Arsha Hita Treatment Group could be a result of the complementary actions of the ingredients present in both Arsha Hita tablets and Arsha Hita ointment. This integrated approach could be considered beneficial in the management of anal fissures.

There are a few limitations to the study's findings that should be considered. Firstly, the study was carried out at a single center, so it may not be representative of the entire patient population. Secondly, the sample size was relatively small since it was a pilot study. Lastly, patients with certain medical conditions were excluded from the study, which limits the applicability of the results to those with these conditions. Overall, further research is necessary to confirm the treatments' safety and effectiveness in a larger and more diverse population of anal fissure patients.

## Conclusions

The present study found that using a combination of Arsha Hita tablets and Arsha Hita ointment was an effective and safe treatment option for anal fissures, with comparable efficacy to standard therapy. The combination treatment was also found to be better in reducing the composite fissure score. However, to further investigate the long-term efficacy of this treatment, a larger follow-up study is recommended. Overall, the results suggest that Arsha Hita tablets and Arsha Hita ointment can be considered as a viable initial treatment option for conservative management of anal fissures.
